# Patient outcomes vs. service workload: an analysis of outcomes in the burn service of England and Wales

**DOI:** 10.1186/s12913-015-0813-4

**Published:** 2015-04-02

**Authors:** Neophytos Stylianou, Matthew Carr, Evangelos Kontopantelis, Iain Buchan, Ken Dunn

**Affiliations:** Centre for Health Informatics, Institute of population Health, University of Manchester, Manchester, UK; Institute of Brain, Behaviour and Mental Health, University of Manchester, Manchester, UK; Institute of Population Health, University of Manchester, Manchester, UK; University Hospital South Manchester and Honorary, Centre for Health Informatics, Institute of Population Health, University of Manchester, Manchester, UK

**Keywords:** Outcome, Burn Injury, LOS, Mortality

## Abstract

**Background:**

Patient outcomes in specialist burns units have been used as a metric of care needs and quality. Besides patient factors there are service factors that might influence Length of Stay (LOS) and mortality, e.g. pressure on beds. Although the bed needs of UK hospitals have dropped significantly over the past three decades, with changes in policies and practices, recent reports suggest that hospitals have 90% bed occupancy for 48 weeks of the year. In the UK, the specialist burn injury service is organised so that patients are assessed on arrival at hospital, and those needing admission are found a nearby bed in a suitable unit through the National Burn Bed Bureau.

The aim of this study was to investigate the effect on outcomes of service pressures due to shortages of beds.

**Methods:**

We took an extract of the anonymised patient data from the specialised burn injury database, iBID, and created a new database based on matching that data with bed availability data provided by the national Burn Bed Bureau. Cox proportional hazard modelling was used for analysis to investigate if there is an impact of bed occupancy (a proxy measure of workload) on LOS.

**Results:**

Cox proportional hazard modelling indicated that half of the services in England and Wales are less likely to discharge a patient if the bed availability is high. Two of the services have abnormally high bed availability and LOS, therefore a model without these two services indicates a general reluctance to discharge patients when beds are available.

**Conclusions:**

It is possible that the effect we observed is a result of gaming as service providers are paid by the number of admissions. In addition, providers many not all give the same level of accuracy of bed availability information to the NBBB: some may under report availability, for example at times of high pressure on staff. Furthermore, burn services may not empty beds to avoid being filled up by work from other specialties, thus making them unable to admit a burn when referred.

## Background

With the current economic crisis, healthcare systems in England and around the world have been burdened with enormous constraints on their expenditure, forcing them to do more with less [1-3]. Pressure on reducing health service budgets has been ongoing since at least the middle of the last century, with suggestions on reducing costs of consumables and increasing efficiency [4]. In the UK, the Department of Health published in 2013 the plan, named Better Care Fund, which is intended to come into action in 2015, aiming at reducing the length of stay of patients and reducing the number of admissions in order to minimise expenditure [5-8].

Historically, decreasing the expenditure of the health system can be achieved by reducing the number of beds in use by hospitals and also the number of patients staying in a hospital bed. To be clear, the decrease in length of stay (LOS) over the years, has other causative factors such as improvements in medical practise, new drugs and diagnostic procedures as well as new policies shifting care from the hospital ward to the outpatients departments and the community [9]. These changes resulted in a total available bed reduction in England of 54% from 1988 to 2013 [10].

The reduction in expenditure and specifically the reduction in available beds needs to be evaluated in respect to the impact it has had on the performance of the system. There are many metrics which can be used in measuring the quality of care and performance of the health service; outcome and process measures. Specifically, for burn services, the outcome measures that are most commonly used are mortality and length of stay.

Mortality is the outcome measure which is traditionally used by burn services, as well as most specialised services [11-13]. Mortality is very well recorded, has a clear and distinct explanation and thus is easily understood by all stakeholders, while a reduction in mortality is probably the best end point. Burn services had dramatically changed the provision of care leading to better mortality outcomes since the end of World War II [14-17]. Mortality in the beginning of the 20^th^ century ranged from 50-100% but current measurements show a range of 4-6% [18-20]. The current low mortality leads to challenging mortality as an outcome measure and drives the need for alternative measures which will be better suited for this kind of injury [21,22].

Various measures have been examined as quality and performance indicators including quality of life and functional status. However, these measures have serious limitations in terms of consistency with respect to age, socioeconomic status and across services [23-27]. On the other hand, length of stay is an outcome measure which has similar characteristics to mortality but does not suffer the imbalance in data associated with low mortality scenarios. Its main drawback is that, due to the nature of the burn injuries, with even minor burns occasionally requiring a long length of stay, the measure is not indicative of burn severity. Length of stay in burn services was originally proposed for the prediction of bed utilisation but more recently it was shown to act as a function of morbidity, complications, and aesthetic and functional outcomes for patients suffering from burn injuries [28-31].

Although major improvements occurred in burn services after World War II, the number and nature of the services largely stagnated until 2001. During that period, planning and organisation of the services was solely reliant on the interests of local burn care teams [32]. In 2001, a review was published which aimed to change the future of burn services in the UK by proposing the centralisation of treatment for burn injuries in sites equipped with specialised multidisciplinary teams. Other important measures recommended in the review included: the differentiation of burn services according to the severity of treated injuries; the establishment of a nationwide database for data collection which would have audit and surveillance purposes, now known as international Burn Injury Database (iBID); the creation of an new body, the National Burn Bed Bureau (NBBB), which would record the number of beds available and then allocate patients according to their needs to the closest available and suitable bed for their severity of injury; referral standards, and many more [32]. This wealth of newly obtained data opens up doors in order to deeply explore the service and suggest possible changes that might make it more efficient and effective.

Investigations into the effect the workload volumes of health services are having on patient outcomes have been performed in the past but have been limited. A systematic review of the literature was conducted by the NHS Centre for Reviews and Dissemination at the University of York. The review suggested that, even if an association between volume and outcome was found, the findings were based on studies that were flawed and inconclusive. As such, they concluded that volume should not be used for service planning and improvement [33]. Since the publication of the systematic review, more studies have investigated the association between volume and outcomes. The studies found that patients treated in high volume hospitals have either unaffected or even improved outcomes [34-42]. We identified only two papers discussing the effect of highly occupied burn services on the outcomes of patients, both of the studies were performed in the United States with both stating that the work had many limitations [43,44].

No research has ever been conducted to investigate the effect of high workload on patient outcomes in the specialised burn service of the National Health Service (NHS) in England and Wales. In this paper, we aim to investigate the effect of occupied burn wards on patient outcomes. More specifically, whether patients get discharged earlier when burn services are full, which may potentially lead to readmission. Another question we will attempt to address is whether there are self-protection practices being employed by services when they are near full capacity, by recording that they are full so that they will not have to admit more patients.

## Methods

Data from all services from March 2005 to November 2011 were obtained from the NBBB with some minor gaps (90 days of the whole time period were not available). These records can be linked to the specialised burn injury database, iBID, with relevant clinical data, for patients in England and Wales. We used an extract of anonymised patient data over the same period from iBID, and created a new database by matching the two source databases on date and service provider. Missing data from the injury database was very low for this extract and study period, with an overall completeness of 95% for all cases across all variables. Most of the variables were fully completed for all records with the exception of TBSA, age and graft number. Therefore we considered a complete case analysis acceptable.

The NBBB provided the total available bed numbers for every day, at both the morning and evening recording times, for each service. However, we chose to compute a bed-availability variable by first finding the number of occupied beds each service had on each day at the two recording times, and deducting the number of occupied beds from the maximum ever recorded occupancy for that service. This approach was selected since there was a large statistical discrepancy between the actual reported bed availability and the one we could observe. The latter was calculated as described above.

In the UK, after the reorganisation of the service with the publication of the National Burn Care Review in 2001, the burn injury service is categorised in a three tier system based on the severity/complexity of the burn injury. Burn facilities which are services that provide care for the injuries that are severe enough to be seen by a specialised service (these were excluded form analysis as injuries treated here are not severe enough to require hospitalisation); burn units which look after more severe injuries and the burn centres that looks after the most complex injuries (with minor exceptions of injuries that occurred in the immediate catchment area) [32].

Services were pseudo-anonymised in order to prevent potential problems which may rise from exposing negative results. In total, 17 specialised services were included in the analysis for this study which excluded burn facilities. Most of the services (11) were mixed occupancy services, meaning that they can admit both adults and children. Three services catered for adults only and 3 services only admitted children. Two of the services that were of no particular difference to other services of the same status (burn unit or burn centre mixed occupancy services) were omitted from the analysis due to abnormal values in their records. One of the services had an abnormally high number of beds as their maximum occupancy beds and available beds. The other service had consistently extremely prolonged LOS (>5% of records had LOS of more than 1 year). All analyses performed on 15 services after excluding the two with potentially data quality issues. The beds in services are dependent on the type and size of the service and they ranged from 8 to 22.

An occupancy variable was added to an existing multivariate logistic regression mortality prediction model to investigate the general effect occupancy variable have on the mortality outcome [45]. The model included the predictor variables of age as a continuous variable, age^2^ as a continuous variable to adjust for non-linearity, TBSA as a continuous variable, injury type as a categorical variable, inhalation injury as a binary variable, existing disorders (which is a database computed variable which counts the existing active disorders of patient upon admission e.g. hypertension, mental health disorders etc.) as a binary variable of <3 and ≥3 and the newly introduced occupancy variable as a continuous variable. We applied the model to the complete dataset and tested the models discrimination and compared it to the original model without the occupancy variable. We used the c-statistic for the area under the curve (AUC) for the discrimination test. We also used the Akaike Information Criterion (AIC) to test the gain in model fit when the occupancy variable is included.

Patient demographics (age, sex), in-hospital mortality and determinants of burn outcomes such as Total Burn Surface Area (TBSA), inhalation injury and the number of grafting operations were included in the model, which aimed in exploring LOS, based on suggestions from a systematic review on the predictive ability of LOS [46]. We also explored, after clinician consultations, other variables present in the database such as bed severity allocation which is a variable based on the bed need of the patient. The variable takes values representing increasing severity where, 1 is a normal surgical bed, 2 is a high dependence bed, and 3 to 5 are intensive care beds. The variable is a static measure of the bed need for the specific patient and it is recorded at the time of admission as the agreed bed level.

We developed a Cox proportional hazards model to investigate the impact of bed availability (a proxy measure of workload) on LOS adjusting for all other factors that could potentially affect the outcome. We stratified our model based on each site, in order to allow the baseline hazards to be different for each site. We also explored the impact of various interaction terms. We constructed different models for adults (≥16 years) and children (<16). The models were built based on a stepwise selection method as well as expert clinical guidance and the models fit were tested using the AIC. All statistical analyses were performed using Stata Version 12 (StataCorp LP, College Station, TX).

## Results

### General description of data

A total of 37,103 records from 15 services across England and Wales were used in the data analysis. All patients were included in the analysis; survived and died. Since mortality was not big (≈1.5%) it does not make much difference to the direction of the effect we observed. Analyses included all patients since, even the patients that actually died, they were still occupying a bed and contributing to the total workload. The quartiles of LOS for patients that died were 25% 2 days, 50% 7 days and 75% 21 days. Although this distribution of LOS is slightly different to the ones that survived, the large numbers of those who survived overshadow that distribution. Males accounted for 63.9% of the patients admitted in that time period. Age ranged from 0 to 110 with mean (SD) age 25.28 (23.72) and 42.85% of patients were aged under 16 years of age on admission and 57.15% aged 16 and over. The median TBSA, a percentage describing the extent of a burn injury on a human body, was 2.0% (IQR = 4.1%, 25^th^ percentile 0.9, 75^th^ percentile 5). Of all patients admitted for a burn injury, 74.44% did not receive a graft, 20.11% received one graft, 2.65% received two and 2.80% received three. Overall, inhalation injury was recorded for 1.23% of patients with 0.20% for children and 2% for adults.

### Effect of bed availability on mortality

Overall mortality was 1.54% of the total number of admissions during the study period. For the model without the occupied beds variable, the AUC for the Receiver Operator Characteristic (ROC) discrimination was 0.97 (95% CI 0.96-0.98). The discrimination was identical for the model including the occupancy variable; 0.97 (95% CI 0.96-0.98). We found the number of occupied beds to be a significant predictor of mortality (see Table 1); the odds ratio for an additional occupied bed was 1.01 [95% CI 1.00, 1.02]. However, the comparison of the AUC of the ROC curves based on the DeLong algorithm [47], demonstrated that the models were not significantly different, p-value = 0.46. We also compared the models using the AIC and found that the inclusion of the occupied beds variable did not significantly improve the fit of the model.

**Table 1:** **Mortality prediction model with number of occupied beds as a predictor**

**Variable**		**Odds ratio**	**95% Confidence intervals**	
**Occupied beds**		1.013	1.004	1.022
**Age**		1.028	1.008	1.048
**Age** ^**2**^		1.000	1.000	1.005
**TBSA**		1.087	1.081	1.093
**Inhalation injury**	Absent	1		
	Present	5.559	4.130	7.481
**Existing disorders**	<3	1		
	> = 3	1.316	1.217	1.424
**Injury mechanism**	Flame	1		
	Flash	0.225	0.119	0.425
	Contact	0.406	0.270	0.612
	Scald	0.563	0.424	0.749
	Chemical	0.118	0.031	0.451
	Other	0.442	0.295	0.664

### Effect of bed availability on LOS of adult patients

We assessed the effect of bed availability by modelling LOS using a Cox proportional hazards model with discharge as the event of interest and LOS as the ‘time-to-event’. We adjusted for all variables recorded in the burns injury database that Hussain and Dunn [46] defined as important predictors of LOS and selected the model with the lowest AIC. We found that the number of available beds had a significant effect on the decision to discharge, from which we can infer that bed availability has an effect on LOS. The discharge hazard ratio for an additional available bed was 0.98 [0.98, 0.99]; see Table 2.

**Table 2:** **Cox proportional hazards regression model for the time-to-discharge in specialised adult burn services in England and Wales**

**Variable**		**Hazard ratio**	**Standard error**	**P > z**	**95% Confidence Interval**	
**Available**		0.982	0.003	0.001	0.976	0.988
**Age**		0.986	0.000	0.001	0.986	0.987
**TBSA (%)**		0.954	0.001	0.001	0.952	0.957
**Existing disorders**	0	Ref				
	1	0.852	0.018	0.001	0.817	0.888
	2	0.721	0.021	0.001	0.681	0.763
	3	0.684	0.028	0.001	0.632	0.741
	4	0.609	0.029	0.001	0.555	0.669
**Injury type**	Flame	Ref				
	Flash	1.211	0.034	0.001	1.146	1.279
	Contact	1.084	0.028	0.002	1.030	1.142
	Scald	1.039	0.025	0.116	0.991	1.089
	Chemical	1.245	0.040	0.000	1.168	1.327
	Other	1.108	0.039	0.003	1.034	1.186
	Unknown	1.098	0.094	0.277	0.928	1.299
**Inhalation injury**	Absent	Ref				
	Present	0.778	0.050	0.001	0.686	0.882
**Activity**	Employment	Ref				
	Transport	0.776	0.058	0.001	0.671	0.897
	Housework	1.155	0.055	0.002	1.053	1.267
	Washing/Bathing	0.623	0.030	0.001	0.566	0.685
	Food preparation	0.968	0.028	0.259	0.915	1.024
	Sleep/Rest	0.847	0.034	0.001	0.784	0.916
	Child play/exploration	1.236	0.141	0.063	0.989	1.546
	Household DIY	1.152	0.043	0.001	1.071	1.240
	Vehicle DIY	1.106	0.080	0.165	0.959	1.275
	Hobby	0.905	0.078	0.249	0.764	1.072
	Sport	1.118	0.226	0.581	0.752	1.661
	Amusement	0.975	0.053	0.646	0.876	1.085
	Socialising	0.925	0.041	0.078	0.849	1.009
	Argument/Fight	0.883	0.058	0.056	0.777	1.003
	No specific activity	0.848	0.031	0.001	0.790	0.911
	Other	0.882	0.026	0.001	0.834	0.934
	Unknown	0.932	0.034	0.050	0.868	1.000
**Total graft number**	0	Ref				
	1	0.593	0.012	0.001	0.570	0.618
	2	0.330	0.015	0.001	0.301	0.362
	3	0.303	0.016	0.001	0.273	0.335
**Bed severity allocation**	1	Ref				
	2	0.757	0.019	0.001	0.721	0.795
	>3	0.625	0.021	0.001	0.584	0.668

Age and TBSA had a significant impact on the decision to discharge patients with older patients and patients with a higher percentage TBSA having a significantly lower hazard of discharge (and therefore longer lengths of stay). The presence of inhalation injury also had a significant effect on the hazard; HR = 0.78 [0.69, 0.88].

Injury mechanism was also found to have an effect on the discharge of patients. In our model we observed that injury mechanisms –Flash, Contact, Scald, Chemical, Other (Friction, Electrical, Radiation) and Unknown causes of burn - when compared to Flame (reference category), increase the hazard of the patient being discharged. All mechanisms indicated the same effect with statistical significance, increasing the hazard ratio, with the exception of Scald and unknown injury mechanism.

Injury during different activities was also explored giving a good model fit and is thus included in the final model. Different activities gave different hazard ratios for the patient being discharged when compared to the activity reference category which was employment. Some of the different activities increased the hazard (Housework, Child play/exploration, Household DIY, Vehicle DIY, Sport) and some decreased the hazard (Transport, Washing/Bathing, Food preparation, Sleep/Rest, Hobby, Amusement, Argument/Fight, no specific activity, Other and Unknown).

Graft number was a significant and strong explanatory variable for the time to discharge. From the model it was clear that the more grafts the patient had during their stay in the hospital, the less likely it was for the patient to be discharged. For example, having one graft reduced the hazard to 0.59 (95% CI: 0.57, 0.62), compared to none. For patients with three grafts, the hazard ratio for discharge decreased was as low as 0.30 (95% CI: 0.27, 0.34).

Bed severity allocation was also included in the model. This variable indicates the level of bed the patient was admitted to. The hazard ratios for the different categories were as expected. The higher the severity of bed allocation, the less likely the patient was to be discharged from the service.

### Effect of bed availability on adult LOS by site

The hazard ratios for an increase of one available bed on the discharge of an adult patient (adjusting for all other variables) are present in Table 3. Most of the services which admit adult patients were found to have a reduced hazard of discharge when beds are available. Not all of the hazard ratios were statistically significant but those for services 2, 5, 6 and 12 were at a 99.5% Confidence Interval level. Availability in service 12 had the biggest effect on the length of stay of the patient; HR = 0.90 (99.5% CI: 0.83, 0.96].

**Table 3:** **Discharge hazard ratios for an extra available bed for every adult specialised burn injury service in England and Wales adjusting for all other variables**

**Service**	**Hazard ratio**	**P > z**	**Confidence 99.5% interval**	
**1**	0.9548	0.065	0.8899	1.0245
**2**	0.9678	0.001	0.9452	0.9909
**3**	1.0133	0.166	0.9866	1.0407
**4**	0.9951	0.617	0.9682	1.0228
**5**	0.9383	0.001	0.8960	0.9825
**6**	0.9688	0.002	0.9412	0.9970
**7**	0.9856	0.197	0.9548	1.0172
**8**	0.9990	0.925	0.9710	1.0280
**9**	0.9844	0.226	0.9491	1.0210
**10**	0.9913	0.311	0.9677	1.0156
**11**	0.9634	0.223	0.8842	1.0497
**12**	0.8958	0.001	0.8328	0.9636

### Effect of bed availability on LOS of children

The model that best described the discharge process included the variables for availability, TBSA, Injury Mechanism, existence of inhalation injury, the graft number, the bed severity allocation and an interaction term of bed severity allocation with number of available beds, as can be seen in Table 4. This model indicates that an extra available bed increases the hazard of discharge of the patient from the service, but the result is not significant. A stronger effect on length of stay was observed for the graft variable. The more grafts the patient had, the less likely the patient was to be discharged; specifically, having three grafts reduced the hazard to 0.29 (95% CI: 0.25, 0.34), compared to none. Burn surface area had a similar effect; the higher the burned surface area, the greater the reduction in the hazard.

**Table 4:** **Cox proportional hazard regression model for discharge time for specialised burn services admitting children in England and Wales**

**Variable**		**Hazard ratio**	**Standard error**	**P > z**	**95% Interval**	
**Available**		1.0037	0.0063	0.5540	0.9915	1.0161
**TBSA (%)**		0.9357	0.0026	0.0000	0.9305	0.9409
**Injury type**	Flame	Ref				
	Flash	1.4917	0.0946	0.0000	1.3174	1.6890
	Contact	1.3631	0.0569	0.0000	1.2561	1.4793
	Scald	1.2452	0.0464	0.0000	1.1574	1.3396
	Chemical	1.2574	0.1083	0.0080	1.0621	1.4887
	Other	1.4443	0.0870	0.0000	1.2834	1.6253
	Unknown	1.1402	0.1170	0.2010	0.9324	1.3942
**Inhalation injury**	Absent	Ref				
	Present	0.5754	0.1108	0.0040	0.3945	0.8391
**Total grafts**	0	Ref				
	1	0.6379	0.0188	0.0000	0.6020	0.6758
	2	0.4122	0.0265	0.0000	0.3635	0.4674
	3	0.2937	0.0223	0.0000	0.2531	0.3408
**Bed severity allocation**	1	Ref				
	2	1.0333	0.0954	0.7230	0.8623	1.2383
	>3	0.9644	0.1546	0.8210	0.7043	1.3205
**Available-bed severity allocation**	2*available	0.9554	0.0135	0.0010	0.9293	0.9823
	>3*available	0.9386	0.0221	0.0070	0.8963	0.9829

*Interaction.

Injury mechanism analysis demonstrated that all other mechanisms are more likely to be discharged earlier when compared to flame. Having an inhalation injury reduced the hazard of discharge by 42.46%. Severity allocation was not statistically significant in this model but the interaction of bed severity allocation with bed availability was. If the bed needed for the patient is a high dependency bed (severity factor 2), and there is an available bed then the patient has a lower hazard of being discharged compared to a patient that needs a surgical bed (severity factor 1). If the patient is in need of an Intensive care bed (severity factor 3), and there is an available bed then the patient has an even lower hazard of being discharged.

The conclusion is that availability does have a statistically significant impact on length of stay but the effect is limited to those patients allocated to bed severity levels 2 and ≥3.

## Discussion

Our findings for adult patients show that most services are keen to keep their patients when they have available beds. An extra available bed in the service increases the probability of the patient staying longer in the hospital. This behaviour by specialised services is in favour of the patient since the nature of the burn injury is such that even minor injuries can require long lengths of stay due to long treatment and rehabilitation times. What is not in the favour of the patient is the fact that the services are under constant pressure to free beds which may lead to premature discharge of the patient. A report in 2012 suggested that hospitals having more than 90% occupancy for 48 weeks of the year raise concerns about patient safety and quality of care [48]. Safety is a measure of quality of care as Lord Darzi has defined it [49]. Ensuring patient safety is one of the biggest health care challenges today [50-52].

Based on our results of the adults’ patients model, there appears to be an effect, albeit small, that services with high workload and a lack of beds are discharging patients earlier. We have identified an overall small effect that having an extra available bed reduces the hazard ratio to 0.98 (95% CI: 0.98, 0.99) for being discharged. In other words having an available bed increases the chances a patient will stay in the hospital by 2.8%. We believe that these decisions are not taken lightly. Beds nowadays have become a valuable, scarce resource. Physicians are required to make decisions based on resource allocation, despite the insufficient training they received on resource allocation [53-55].

Our results suggest that for a small number of services, bed availability was a significant predictor for LOS, highlighting a potential problem that warrants further investigation. Although the overall effects we identified were quite small, they are still relevant and, having the benefit of patients’ in focus, they should raise concerns regarding discharge practices and the number of beds allocated to burn injury.

In performing our analysis we decided that it was best to use a computed available beds variable in our model instead of using what was recorded by the NBBB. This is because we came to the conclusion that services may choose to record their bed availability based on the pressure they were under at the time. On many occasions we found that the computed available variable did not agree with what was recorded by the bed bureau and that difference was statistically significant.

This inconsistency may have occurred in services when they wanted to provide the best possible care to the patients they were already treating in the service. By underreporting availability to the bed bureau, the bureaus’ triage system would show full occupancy for that service and an inability to admit more patients, thus the service is not jeopardising the quality of care provided. According to Tavaglione and Hurst, 2012, physicians ought to lie for their patients [56]. In their paper they conclude that since care is provided in a non-ideal health system then what is termed as ‘gaming’ is justified in favour of the physician, provided that gaming takes a form in which the Hippocratic duties of the physician are applied.

Since 2000, the NHS has created many measurement systems and indicators aimed at comparing performance in the broad field of health care and between services [57]. In recent years they have focused on providing financial incentives which would be reflecting the service performance and quality of care [58]. According to Mannion [59], these payments for performance can lead to unwanted consequences such as poor measurement and most importantly breaches of trust by gaming. Gaming in healthcare can have both positive and negative properties, for example performing easier/safer surgical procedures to get more funding from the healthcare system or what was previously mentioned, lying for the benefit of the patient.

Therefore, the potential for “early” discharge we can infer from our findings, financial motives may be unearthed. Clinicians may take the decision to discharge patients earlier when there is need for a bed by another patient in order to receive another admission. They may also arrange for the later readmission of the first patient, thus increasing the admissions of their service, which in turn will reflect on their financial reimbursement. This may be an extreme scenario but is one that reflects the perverse incentives the current arrangements are capable of promoting.

Contrary to the adult model the model for children has showed a hazard ratio of 1.004 for an increase of one available bed adjusting for all other variables, although that value is not statistically significant. The results indicate that availability has an increasing impact on length of stay for increasing severity of bed allocation. The care of children in a burn service appears to differ greatly from the care adults receive. An explanation for this could be the fact that age has an important role in the mechanism of burn injury, thus severity. Children usually suffer scald burns which although serious are usually not life threatening and can be more easily dealt as outpatients with the proper parent education and support.

Another explanation for the difference in care of adults and children could be attributed to biological reasons due to age. It has been extensively discussed that advancing age has an effect on adverse complications and outcome of patient [45,60,61] indicating that the human body when younger is able to cope and overcome the injury easier. In a recent study we performed, we discovered that children contribute around 40% of the workload of attendances and admissions to the specialised burn services but their LOS is mostly one day, while for adults and the elderly, it increases significantly [62]. This short length of stay could be attributed to the precautionary behaviour the health system takes with regard to children due to their young age as well as protecting the child if the suspected cause of the injury is parental abuse.

### Limitations of the study

Workload could be more completely measured by incorporating the levels of staff found in the burn service in our analysis. This would give a more complete picture of the workload since understaffed wards can also play an important role on the outcome of patients.

We used an approximation of the available beds found in the service. Bed allocations change occasionally in the specialised burn services therefore increasing or decreasing maximum occupancy capabilities. We took a static measurement of available beds by computing them for the maximum occupancy the services ever had and deducted the number of patients they recorded in the injury database during the specific time of that particular day.

A holistic service effect could be investigated if we did not have to exclude two of the services from our analyses due to data quality issues. One of the services had an unusual maximum bed allocation (exceeding 35) and the other had extreme patient lengths of stay. Iterative improvements in data quality should allow these services to be included in future analysis.

## Conclusions

Our study provides for the first time evidence relating to workload’s effect on outcomes of the patients. Our study can be considered as an initial step to investigate the correct number of beds a service should have. We have indicated the importance bed availability has on length of hospitalisation of patients, thus indicating the correct placement of beds will play an important role in the care the patients will receive as well as more efficient and effective resource allocation.

It is well documented that using process measures has advantages over outcome measures in performance measurement [63-65]. We proved with our analysis that we can use routinely collected data to create a hybrid model which uses outcome measures to investigate clinical processes in order to measure performance.

We are strong advocates that judgment and penalisation is not an appropriate form of behaviour change and other more efficient behaviour change models should be applied in the healthcare sector. Since our target is service improvement we have not used service names as we did not want to judge services but wish to understand how services might reach the desired levels of high quality healthcare provision.

One way of supporting services in achieving higher quality of care, thus improving the service all together, is by helping them realise that high quality data is a necessity. Achieving that will allow clearer indications of where problems exist, the magnitude of the problems and what interventions could be tailored and directly targeted to those specific areas. Our study could not be achieved if we did not have this wealth of data, nonetheless there is still room for improvement. We identified the fact that there is a potential problem with allocated beds which may have an impact on the quality of care provided, thus more cautious service planning is necessary in order to avoid forfeiting quality of care and service improvement by being too rigid about the number of beds necessary to provide a high quality service at all times.

### Ethical approval

Not needed as no identifiable information were used.
